# Endocrine disruptors and bladder function: the role of phthalates in overactive bladder

**DOI:** 10.3389/fpubh.2024.1493794

**Published:** 2024-12-11

**Authors:** Li Liu, Xia Li, Xuexue Hao, Zhunan Xu, Qihua Wang, Congzhe Ren, Muwei Li, Xiaoqiang Liu

**Affiliations:** Department of Urology, Tianjin Medical University General Hospital, Tianjin, China

**Keywords:** OAB, phthalates, endocrine-disrupting chemicals, NHANES, cross-sectional study

## Abstract

**Background:**

Phthalates, widely used as plasticizers, are pervasive environmental contaminants and endocrine disruptors. Their potential role in overactive bladder (OAB) pathogenesis is underexplored, necessitating further investigation into their impact on OAB using large-scale epidemiological data.

**Methods:**

This study utilized data from the National Health and Nutrition Examination Survey (NHANES) spanning from 2011 to 2018. A weighted multivariable logistic regression model was employed to examine the relationship between urinary phthalate concentrations and OAB. Subgroup analyses were conducted to explore differences in associations across various subgroups. Restricted cubic spline (RCS) analysis was used to investigate the potential non-linear relationship between urinary phthalate concentrations and OAB. Additionally, Bayesian Kernel Machine Regression (BKMR) analysis was performed to explore the overall effects and interactions of phthalate mixtures.

**Results:**

In the multivariable logistic regression model fully adjusted for confounding variables, higher concentrations of MBzP and MiBP were associated with an increased risk of OAB, particularly in the highest tertiles (MBzP: OR = 1.401, 95% CI: 1.108–1.771; MiBP: OR = 1.050, 95% CI: 1.045–1.056). Subgroup analysis found that subgroup characteristics did not have a significant moderating effect on the association between phthalates and OAB. RCS analysis revealed a linear relationship between both MBzP and MiBP and OAB. BKMR analysis confirmed a positive overall effect of phthalate mixtures on OAB risk, with MBzP identified as the major contributing factor.

**Conclusion:**

In our study cohort, a positive correlation between urinary phthalate concentrations and OAB was observed, necessitating further research to validate and refine this conclusion.

## Introduction

Phthalates, a class of synthetic chemicals extensively employed as plasticizers and constituents of personal care products, have garnered significant attention due to their pervasive environmental presence and potential health-disrupting effects (1). As phthalates do not form covalent bonds when mixed with plastics, they are easily released into the environment and leach from consumer products as plastics age and degrade, leading to widespread human exposure (2). Phthalates are primarily absorbed via ingestion, inhalation, and dermal contact, with vapor-phase phthalates representing a significant route of exposure through skin absorption (3). Furthermore, these compounds have been detected in diverse human tissues and biological fluids, including urine and blood (4, 5). The potential health risks posed by phthalate exposure have attracted growing concern, especially due to their function as endocrine disruptors (6). These compounds disrupt hormonal regulation and are implicated in a range of adverse health effects, including reproductive toxicity (7), developmental abnormalities (8), and carcinogenic effects (9, 10).

Overactive bladder (OAB) is a widespread urological disorder marked by symptoms including urinary urgency, frequency, and nocturia, which may occur with or without urgency incontinence. OAB afflicts millions globally, significantly diminishing quality of life and resulting in physical, emotional, and social challenges (11). Although OAB is highly prevalent, its pathophysiology is not yet fully elucidated. Studies indicate a complex interaction among factors related to the nervous system, bladder smooth muscle, and urothelial cells (12). While genetic predisposition and lifestyle factors have been investigated, environmental exposures, particularly endocrine-disrupting chemicals such as phthalates, are increasingly considered potential contributors to the onset and progression of OAB (13).

Earlier research has examined the impact of phthalates on multiple dimensions of human health, with some studies highlighting their detrimental effects on the urinary system, including associations with prostate cancer, bladder cancer, and testicular toxicity (14–16). The study by Yang et al. demonstrated that exposure to phthalates may elevate the risk of overactive bladder; however, it utilized outdated data (13). Comprehensive, population-based research employing large-scale epidemiological data is required to clarify the potential association between phthalate exposure and OAB. The objective of this study is to evaluate the relationship between phthalate exposure and OAB utilizing data from National Health and Nutrition Examination Survey (NHANES) 2011–2018. By capitalizing on this extensive and representative dataset, we aim to ascertain whether phthalate exposure serves as a significant risk factor for OAB and may contribute to the heightened burden of this condition within the population.

## Materials and methods

### Data source and study population

This study draws on data from the 2011–2018 NHANES, a cross-sectional survey intended to evaluate the health and nutritional status of the civilian, non-institutionalized U.S. population. The survey employs a complex, multistage probability sampling design to ensure representativeness of the U.S. population. Given the extensive scope of NHANES data, including detailed urinary biomarker measurements of phthalate metabolites, it provides a unique opportunity to investigate the potential association between phthalate exposure and OAB on a broad scale.

**Figure 1:**
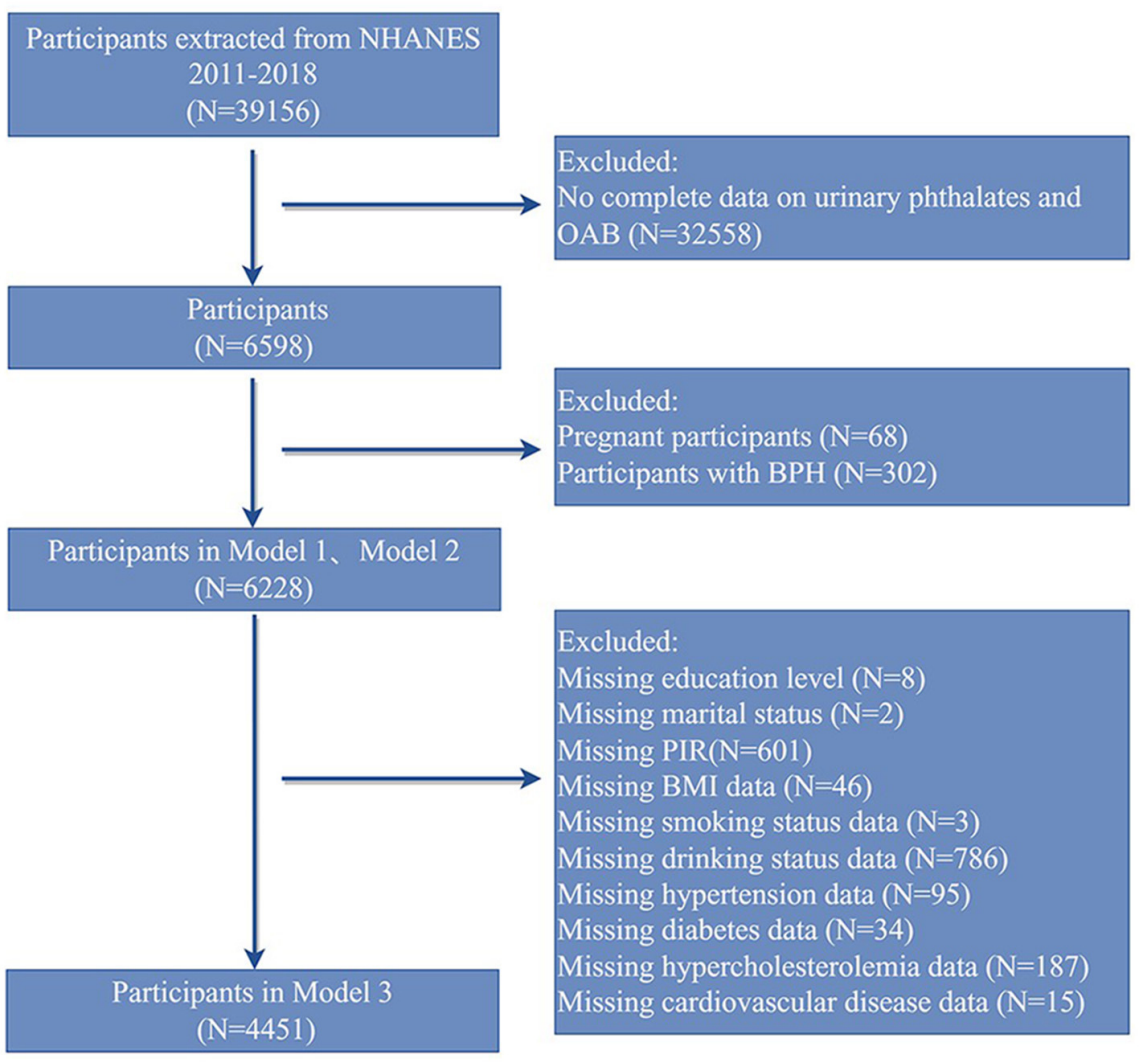
The flowchart for participant screening in this study.

The study cohort comprised adults aged 20 years and above who participated in NHANES between 2011 and 2018 and had complete data on urinary phthalate metabolites and OAB assessments. Based on this, individuals who were pregnant or had a documented history of known diseases affecting bladder function were first excluded. Ultimately, 6,228 participants were included in Models 1 and 2. Then, individuals with missing covariate data were excluded, resulting in a final inclusion of 4,451 participants in Model 3 (Figure 1).

### Assessment of urinary phthalate concentrations

Isotope dilution high-performance liquid chromatography-tandem mass spectrometry (HPLC-MS/MS) was employed for the quantitative analysis of urinary phthalate metabolites. Urine was processed using β-Glucuronidase for the digestion of glucurnide-conjugated metabolites, followed by online solid-phase extraction (SPE) coupled with reverse-phase HPLC-ESI-MS/MS. Detailed experimental methods can be found in the NHANES 2017–2018 Laboratory Methods. Urine samples were collected from participants and analyzed for a range of phthalate metabolites, including high-molecular-weight (HMW) phthalates such as mono(carboxynonyl) phthalate (MCNP), mono(carboxyoctyl) phthalate (MCOP), mono-2-ethyl-5-carboxypentyl phthalate (MECP), mono(3-carboxypropyl) phthalate (MCPP), and monobenzyl phthalate (MBzP). Low-molecular-weight (LMW) phthalates, such as monoethyl phthalate (MEP), monobutyl phthalate (MBP), and mono-isobutyl phthalate (MiBP), were also included. The selection of these metabolites was based on their prevalence in the general population and their potential health implications (17, 18). Urinary creatinine levels were concurrently measured to adjust for urine dilution in the analyses, detailed experimental methods can be found in the NHANES 2017–2018 Laboratory Methods. Quality control procedures involved the use of blanks, calibration standards, and quality control samples with known concentrations of phthalate metabolites. All laboratory analyses were conducted by the Laboratory Sciences Division of the Centers for Disease Control and Prevention (CDC).

### Assessment of OAB

The Overactive Bladder Symptom Score (OABSS) is a standardized tool developed to quantify the severity of symptoms in patients with OAB (19). Urinary symptom questionnaire data from the NHANES survey were translated into OABSS scores to determine the presence of OAB in study participants (20, 21) (Table 1). Participants were asked: how often have you experienced urinary leakage? A score of 0 on the OABSS corresponded to “Never,” while “Less than once a month” and “A few times a month” were both scored as 1. “A few times a week” was assigned a score of 2, and “Every day or night” was scored as 3. Nocturia was evaluated in a similar manner, with scores assigned based on the frequency of nocturnal awakenings to urinate. “0 times” corresponded to an OABSS score of 0, “1 time” to a score of 1, and “2 times” to a score of 2. The total OABSS was derived by summing the scores across these categories, providing a quantitative measure of OAB symptom severity for each participant. Individuals with a total score of ≥3 were classified as having OAB (22). This scoring approach provides a standardized evaluation of OAB within the study cohort, thereby enhancing comparability and supporting rigorous statistical analysis. In addition, we performed a sensitivity analysis using an OABSS threshold of ≥5 to assess the robustness of our main findings.

**Table 1:** Criteria for conversion of symptom frequencies recorded in NHANES and OABSS scores.

**NHANES score**	**OABSS score**
**How often have urinary leakage**	**Urge urinary incontinence score**
Never	0
Less than once a month	1
A few times a month	1
A few times a week	2
Every day or night	3
**How many times urinate in night**	**Nocturia frequency Nocturia score**
0	0
1	1
2	2
3	3
4	3
5 or more	3

OABSS, Overactive Bladder Symptom Score.

### Covariates

Our analysis included a range of covariates to account for potential confounders. The selection of these covariates was guided by their established or hypothesized relationships with phthalate exposure and OAB (23, 24). The covariates considered in the analysis included age (years), gender (male or female), race/ethnicity, body mass index (BMI, kg/m^2^), educational attainment, socioeconomic status, smoking status (current, former, never), alcohol consumption (light, moderate, or heavy), hypertension, diabetes, hyperlipidemia, and a history of cardiovascular disease. BMI, hypertension, diabetes, and hyperlipidemia were evaluated using physical examination data, while the other covariates were obtained from self-reported questionnaire data.

### Statistical analysis

All statistical analyses were conducted using Stata 17 and R version 4.3.1. Survey design variables and sample weights were integrated into all analyses, with MEC weights adjusted by a factor of 0.25 to yield final weights for the four survey cycles spanning 2011 to 2018. MEC weight refers to the weight assigned to survey participants based on the Mobile Examination Center data. Descriptive statistics were utilized to summarize the demographic characteristics of the study cohort. Categorical variables were represented using weighted proportions and 95% confidence intervals (95% CI), while continuous variables were presented using weighted means and standard errors (SE). We used SPSS to conduct Pearson chi-squared tests for categorical variables and *t*-tests for continuous variables to assess group differences. The association between urinary phthalate metabolite concentrations and OAB was assessed using multivariate logistic regression models, with subgroup analyses performed to further investigate the findings.

To investigate the potential linear or non-linear associations between urinary phthalate concentrations and OAB, a restricted cubic spline (RCS) analysis was employed. The RCS method enables flexible modeling of non-linear relationships without requiring the imposition of a specific functional form. The results were subsequently visualized to offer a comprehensive understanding of the dose-response relationship (25).

We applied a Bayesian Kernel Machine Regression (BKMR) model to evaluate the potential interactions among various phthalate metabolites and their collective impact on OAB. The BKMR approach is particularly effective for studies involving multiple exposure mixtures, as it enables the assessment of compound interactions and their combined effects on OAB risk (26).

## Results

### Characteristics of study participants

The baseline characteristics of the study participants are detailed in Table 2. The study included 4,451 participants, with an average age of 46.46 years (SE = 0.33). Compared to participants without OAB, those with OAB were more likely to be older, predominantly female, and have a higher BMI (*P* < 0.001). Compared to the non-OAB population, the proportion of individuals with hypertension, diabetes, and cardiovascular diseases is higher in the OAB population (*P* < 0.001), while there is no difference in the proportion of high cholesterol between the two groups (*P* > 0.05). Furthermore, compared to non-OAB participants, individuals with OAB exhibited elevated average concentrations of specific phthalate metabolites, such as MBzP and MiBP (9.12 vs. 7.94; 15.67 vs. 13.53; *P* < 0.05), while the concentration of MCOP was found to be lower in participants with OAB (27.29 vs. 37.93; *P* < 0.001). Compared to the non-OAB population, the proportion of smokers is higher and the proportion of drinkers is lower in the OAB population (*P* < 0.001).

**Table 2:** General baseline characteristics of the participants by overactive bladder or non-overactive bladder in the NHANES 2011–2018^a^.

**Characteristic**	**Total**	**Non-overactive bladder**	**Overactive bladder**	***P*-value**
	**(*****n*** = **4,451)**	**(*****n*** = **3,416)**	**(*****n*** = **1,035)**	
Age (years), mean (SE)	46.46 ± 0.33	43.96 ± 0.36	56.47 ± 0.61	<0.001
**Sex (%)**				<0.001
Male	50.23 (48.20, 52.25)	54.06 (51.78, 56.33)	34.89 (30.76, 39.27)	
Female	49.77 (47.75, 51.80)	45.94 (43.67, 48.22)	65.11 (60.73, 69.24)	
**Race (%)**				<0.001
Non-Hispanic White	68.12 (66.54, 69.66)	68.08 (66.3, 69.81)	68.29 (64.84, 71.55)	
Non-Hispanic Black	10.50 (9.76, 11.29)	9.74 (8.94, 10.60)	13.56 (11.76, 15.58)	
Mexican American	7.77 (7.06, 8.54)	7.96 (7.15, 8.85)	7.01 (5.73, 8.54)	
Non-Hispanic Asian	4.63 (4.21, 5.10)	5.11 (4.61, 5.67)	2.72 (2.09, 3.53)	
Hispanic	5.74 (5.13, 6.41)	5.77 (5.08, 6.54)	5.61 (4.41, 7.12)	
Other Race	3.23 (2.63, 3.98)	3.34 (2.67, 4.17)	2.81 (1.61, 4.89)	
**Educational level (%)**				<0.001
Below high school	11.62 (10.66, 12.65)	10.59 (9.56, 11.72)	15.70 (13.42, 18.29)	
High school	22.09 (20.43, 23.85)	21.87 (20.01, 23.86)	22.94 (19.46, 26.85)	
Some college or AA degree	32.27 (30.44, 34.15)	31.64 (29.61, 33.74)	34.78 (30.76, 39.04)	
College graduate or above	34.03 (32.04, 36.08)	35.89 (33.64, 38.21)	26.57 (22.50, 31.08)	
**Marital status (%)**				0.016
Married or living with a partner	36.96 (35.07, 38.90)	36.79 (34.65, 38.98)	37.65 (33.67, 41.81)	
Living alone	63.04 (61.10, 64.93)	63.21 (61.02, 65.35)	62.35 (58.19, 66.33)	
**Socioeconomic status (%)**				<0.001
Low	20.49 (19.17, 21.88)	19.19 (17.73, 20.73)	25.7 (22.63, 29.02)	
Moderate	33.33 (31.51, 35.2)	32.43 (30.40, 34.52)	36.95 (32.91, 41.19)	
High	46.18 (44.12, 48.25)	48.38 (46.08, 50.69)	37.35 (32.87, 42.05)	
**BMI (%)**				<0.001
<25 kg/m^2^	29.53 (27.73, 31.41)	32.03 (29.94, 34.19)	19.54 (16.44, 23.07)	
25–30 kg/m^2^	32.42 (30.53, 34.37)	33.03 (30.91, 35.23)	29.96 (25.93, 34.33)	
≥30 kg/m^2^	38.05 (36.10, 40.03)	34.94 (32.79, 37.15)	50.50 (46.11, 54.88)	
**Smoking status (%)**				<0.001
Never	57.88 (55.86, 59.86)	59.04 (56.77, 61.28)	53.20 (48.83, 57.52)	
Former	23.09 (21.38, 24.90)	22.24 (20.33, 24.27)	26.51 (22.82, 30.56)	
Current	19.03 (17.56, 20.59)	18.72 (17.06, 20.49)	20.29 (17.26, 23.70)	
**Alcohol intake (%)**				0.001
Low	10.42 (9.38, 11.56)	9.49 (8.41, 10.68)	14.17 (11.44, 17.41)	
Moderate	39.75 (37.75, 41.78)	39.78 (37.53, 42.07)	39.61 (35.33, 44.06)	
High	49.83 (47.8, 51.86)	50.74 (48.45, 53.02)	46.22 (41.90, 50.60)	
**Hypertension (%)**				<0.001
No	85.76 (84.33, 87.09)	87.57 (86.01, 88.98)	78.54 (74.85, 81.81)	
Yes	14.24 (12.91, 15.67)	12.43 (11.02, 13.99)	21.46 (18.19, 25.15)	
**Diabetes (%)**				<0.001
No	89.82 (88.59, 90.93)	92.08 (90.77, 93.22)	80.78 (77.43, 83.73)	
Yes	10.18 (9.07, 11.41)	7.92 (6.78, 9.23)	19.22 (16.27, 22.57)	
**Hypercholesterolemia (%)**				0.366
No	88.48 (87.05, 89.77)	88.87 (87.26, 90.30)	86.91 (83.58, 89.64)	
Yes	11.52 (10.23, 12.95)	11.13 (9.70, 12.74)	13.09 (10.36, 16.42)	
**Cardiovascular disease (%)**				<0.001
No	92.69 (91.65, 93.62)	94.67 (93.58, 95.59)	84.77 (81.73, 87.39)	
Yes	7.31 (6.38, 8.35)	5.33 (4.41, 6.42)	15.23 (12.61, 18.27)	
**High molecular phthalates (ng mL** ^−1^ **) mean (SE)**				
MCNP	4.43 ± 0.43	4.2 ± 0.26	5.33 ± 1.88	0.733
MCOP	35.8 ± 1.85	37.93 ± 2.19	27.29 ± 2.94	<0.001
MECP	12.25 ± 0.5	12.23 ± 0.59	12.32 ± 0.88	0.062
MCPP	5.93 ± 0.61	6.05 ± 0.72	5.46 ± 0.95	0.610
MBzP	8.18 ± 0.32	7.94 ± 0.36	9.12 ± 0.71	0.031
**Low molecular phthalates (ng mL**−**1) mean (SE)**				
MEP	147.31 ± 12.73	146.5 ± 14.81	150.56 ± 23.26	0.702
MBP	16.03 ± 1.07	16.05 ± 1.31	15.96 ± 1.03	0.847
MiBP	15.24 ± 0.59	13.53 ± 0.67	15.67 ± 0.72	0.040
OABSS score, mean (SE)	1.49 ± 0.03	0.92 ± 0.02	3.73 ± 0.04	<0.001

^a^Participants included in Model 3.

HMW, high molecular weight; LMW, low molecular weight; MCNP, mono(carboxynonyl)phthalate; MCOP, mono(carboxyoctyl) phthalate; MECP, Mono-2-ethyl-5-carboxypentyl phthalate; MCPP, mono-(3-carboxypropyl) phthalate; MBzP, mono-benzyl phthalate; MEP, mono-ethyl phthalate; MBP, mono-n-butyl phthalate; MiBP, mono-isobutyl phthalate.

### Association between the urinary phthalate concentrations and OAB

The association between urinary phthalate concentrations and the risk of OAB was assessed using multivariable logistic regression models (Table 3). After adjusting for potential confounders, we observed that when phthalate concentrations were treated as continuous variables, higher concentrations of HMW phthalate MBzP were associated with an increased risk of OAB. This association was more pronounced in the highest tertile range when phthalate concentrations were treated as categorical variables (OR = 1.401, 95% CI: 1.108, 1.771). Likewise, an increase in LMW phthalate MiBP levels was correlated with a higher OAB risk, especially in the highest tertile (OR = 1.050, 95% CI: 1.045, 1.056). When using the OABSS threshold of ≥5 to define OAB, MBzP, and MiBP still showed an association with OAB in model 3, after adjusting for all covariates, especially in the higher percentiles (OR = 1.509, 95% CI: 1.041, 2.186; OR = 1.555, 95% CI: 1.088, 2.224). The results of this sensitivity analysis are provided in the Supplementary Table 1.

**Table 3:** The weighted multivariate logistic regression analysis of the relationship between phthalates exposures and overactive bladder.

**HMW phthalates**	**Model 1^a^ (*n* = 6,228)**	**Model 2^b^ (*n* = 6,228)**	**HMW phthalates**	**Model 3^c^ (*n* = 4,451)**
	**OR (95% CI)**	**OR (95% CI)**		**OR (95% CI)**
**MCNP**	0.999 (0.995–1.004)	1.001 (0.997–1.005)	**MCNP**	1.002 (0.998–1.006)
Q1 (<1.1)	Reference	Reference	Q1 (<1.2)	Reference
Q2 (1.1–2.7)	**1.212 (1.024–1.434)** ^*^	1.211 (0.977–1.500)	Q2 (1.2–2.8)	1.223 (0.979–1.528)
Q3 (>2.7)	0.901 (0.756–1.074)	1.083 (0.833–1.409)	Q3 (>2.8)	1.058 (0.808–1.386)
**MCOP**	**0.998 (0.997–0.999)** ^**^	0.999 (0.998–1.000)	**MCOP**	1.000 (0.998–1.001)
Q1 (<5.2)	Reference	Reference	Q1 (<5.5)	Reference
Q2 (5.2–17.47)	0.974 (0.823–1.152)	0.818 (0.659–1.017)	Q2 (5.5–18.6)	0.783 (0.626–1.002)
Q3 (>17.47)	0.843 (0.710–1.002)	0.827 (0.623–1.098)	Q3 (>18.6)	0.782 (0.587–1.042)
**MECP**	0.998 (0.996–1.001)	0.998 (0.996–1.001)	**MECP**	0.997 (0.993–1.000)
Q1 (<6.5)	Reference	Reference	Q1 (<6.4)	Reference
Q2 (6.5–14.7)	**1.195 (1.007–1.418)** ^*^	1.172 (0.942–1.458)	Q2 (6.4–14.6)	1.106 (0.884–1.382)
Q3 (>14.7)	1.099 (0.924–1.306)	1.081 (0.821–1.422)	Q3 (>14.6)	0.914 (0.698–1.196)
**MCPP**	1.000 (0.998–1.001)	1.000 (0.999–1.002)	**MCPP**	1.000 (0.999–1.002)
Q1 (<0.9)	Reference	Reference	Q1 (<0.9)	Reference
Q2 (0.9–2.4)	1.097 (0.925–1.301)	0.987 (0.791–1.233)	Q2 (0.9–2.4)	1.035 (0.824–1.299)
Q3 (>2.4)	0.983 (0.828–1.166)	1.051 (0.793–1.394)	Q3 (>2.4)	1.156 (0.867–1.542)
**MBzP**	**1.005 (1.001–1.009)** ^*^	**1.007 (1.003–1.011)** ^***^	**MBzP**	**1.005 (1.001–1.008)** ^*^
Q1 (<2.2)	Reference	Reference	Q1 (<2.1)	Reference
Q2 (2.2–6.4)	1.067 (0.898–1.268)	1.093 (0.889–1.344)	Q2 (2.1–6.3)	0.993 (0.803–1.228)
Q3 (>6.4)	1.140 (0.960–1.354)	**1.329 (1.041–1.695)** ^*^	Q3 (>6.3)	**1.401 (1.108–1.771)** ^**^
**LMW phthalates**			**LMW phthalates**	
**MEP**	1.000 (1.000–1.000)	1.000 (1.000–1.000)	**MEP**	1.000 (1.000–1.000)
Q1 (<19.7)	Reference	Reference	Q1 (<19.5)	Reference
Q2 (19.7–68.6)	0.924 (0.776–1.099)	0.824 (0.677–1.003)	Q2 (19.5–67.9)	0.808 (0.659–1.110)
Q3 (>68.6)	**1.188 (1.004–1.405)** ^*^	1.060 (0.865–1.299)	Q3 (>67.9)	0.944 (0.769–1.158)
**MBP**	1.000 (0.998–1.001)	0.999 (0.997–1.001)	**MBP**	0.999 (0.997–1.001)
Q1 (<6.3)	Reference	Reference	Q1 (<6.1)	Reference
Q2 (6.3–15.4)	1.168 (0.982–1.389)	1.111 (0.875–1.409)	Q2 (6.1–15.3)	1.179 (0.924–1.504)
Q3 (>15.4)	**1.266 (1.067–1.503)** ^**^	1.171 (0.869–1.578)	Q3 (>15.3)	1.281 (0.950–1.728)
**MiBP**	**1.004 (1.001–1.007)** ^*^	**1.004 (1.002–1.007)** ^**^	**MiBP**	**1.004 (1.001–1.007)** ^*^
Q1 (<4.9)	Reference	Reference	Q1 (<4.8)	Reference
Q2 (4.9–11.9)	0.984 (0.830–1.168)	0.930 (0.741–1.167)	Q2 (4.8–11.9)	0.843 (0.668–1.065)
Q3 (>11.9)	0.992 (0.837–1.176)	**1.048 (1.043–1.053)** ^***^	Q3 (>11.9)	**1.050 (1.045–1.056)** ^*^

^a^Model 1: no adjusted.

^b^Model 2: adjusted for age, sex.

^c^Model 3: adjusted for all covariates.

^*^p < 0.05, ^**^p < 0.01, ^***^p < 0.001. Bold values indicate statistical significance.

### Subgroup analysis

**Figure 2:**
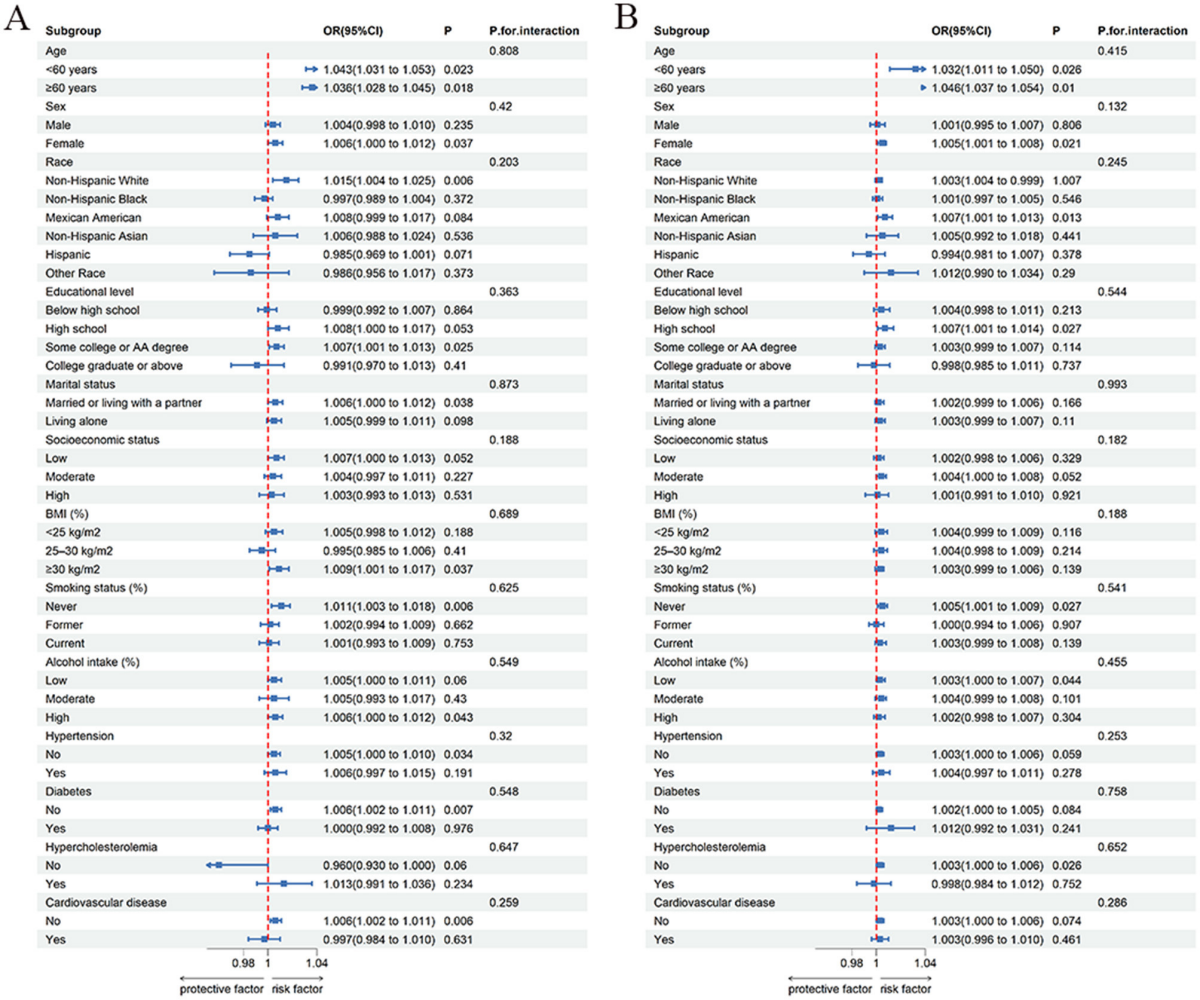
Subgroup analysis of phthalates exposures and overactive bladder. **(A)** MBzP; **(B)** MiBP; Each group includes adjustment for all covariates except for the grouping factor.

To explore whether the association between phthalate exposure and OAB varies across age, sex, and other covariates, we performed subgroup analyses of MBzP and MiBP (Figure 2). The results indicate that no significant interaction was found across all subgroups (*P* > 0.05), suggesting that the effects of MBzP and MiBP concentrations on OAB are consistent among these subgroups, and subgroup characteristics did not have a significant moderating effect on the associations between MBzP and OAB, as well as MiBP and OAB.

### RCS analysis

**Figure 3:**
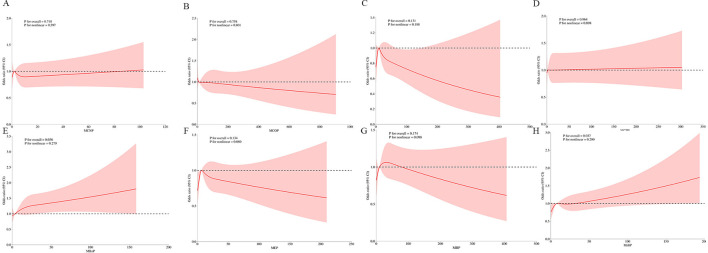
Weighted RCS analysis of the association between phthalates exposures and overactive bladder. **(A)** MCNP; **(B)** MCOP; **(C)** MECP; **(D)** MCPP; **(E)** MBzP; **(F)** MEP; **(G)** MBP; **(H)** MiBP. The model adjusted for all covariates.

We conducted a RCS analysis to assess the potential non-linear relationship between urinary phthalate concentrations and OAB risk (Figure 3). The results indicated a positive correlation between MBzP and MiBP with OAB (*P* for overall < 0.05), with the relationship being linear (*P* for non-linear > 0.05).

### BKMR analysis

**Figure 4:**
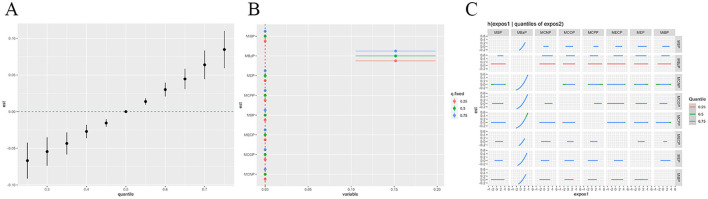
**(A)** Mixture overall effect in BKMR analysis; **(B)** single-component effect in BKMR analysis; **(C)** multivariable exposure-response interaction in BKMR analysis; The model adjusted for all covariates. est: overall risk estimate for mixed exposure; quantile: percentile ranges of mixed exposure.

BKMR analysis revealed an overall positive impact of phthalate mixtures on OAB risk, indicating that higher levels of combined exposure are associated with an increased risk of OAB. The results of the BKMR model indicate that MBzP has the highest probability of inclusion (*P* = 1.00), suggesting that MBzP may be the primary factor contributing to this effect. The joint and individual effects of phthalate metabolites on OAB risk as revealed by the BKMR analysis are presented in Figures 4A, B. Furthermore, the analysis explored potential interactions between different phthalate exposures, but no significant interactions were found (Figure 4C).

## Discussion

In this study, we leveraged data from NHANES 2011–2018 to investigate the association between urinary phthalate concentrations and the risk of OAB. Our findings indicate that higher concentrations of HMW phthalate metabolite MBzP and LMW phthalate metabolite MiBP are associated with an increased risk of OAB. These associations were particularly pronounced in the highest tertile of phthalate exposure. Subgroup analysis found that subgroup characteristics did not have a significant moderating effect on the associations between MBzP and OAB, as well as MiBP and OAB. Furthermore, RCS analysis indicated a linear relationship between these phthalates and OAB risk, while BKMR analysis identified MBzP as the primary contributor to the overall positive effect of the phthalate mixture on OAB risk.

Our findings are consistent with previous research, suggesting that phthalates may exert detrimental effects on bladder function. Specifically, di-n-butyl phthalate (DBP) has been shown to promote bladder cancer progression through the induction of gene alterations (15), DBP can be metabolized in the body to form MBzP and MiBP. Beyond the bladder, phthalates may also have a range of harmful effects on the urinary system. For instance, phthalates have been linked to kidney damage through mechanisms involving PINK1/Parkin-mediated mitophagy and mitochondrial energy deficiency (27). Additionally, phthalate exposure has been associated with an increased risk of urinary incontinence and the induction of prostatic hyperplasia (17, 28). Multiple studies have also identified a strong association between phthalate exposure and the incidence and progression of various urinary system tumors (29, 30). Furthermore, our study identified a significant association between MBzP and MiBP and OAB, while the other six phthalates did not demonstrate such correlations. Considering the substantial differences in MBzP and MiBP levels between the OAB and non-OAB groups within the study population, we propose that the concentration differences of other phthalates may be insufficient to indicate a statistically significant association with OAB. Our findings further reveal a stronger association between MBzP and OAB within the higher percentile ranges. Previous studies have also indicated that MBzP tends to exhibit significant associations with other diseases among phthalates (31, 32), which may be attributed to its known reproductive toxicity (33).

The biological mechanisms underlying phthalate exposure and OAB may be exceedingly complex, encompassing multiple dimensions. Existing relevant research is relatively limited and has yet to elucidate these specific mechanisms comprehensively. One of the endocrine disruptors, phthalates are well-known for their ability to interfere with normal hormonal regulation. Research indicates that exposure to phthalates can lead to significant alterations in thyroid hormone levels (34). Furthermore, studies have found that phthalate exposure is negatively correlated with the estrogen/testosterone ratio (35). Estrogen plays a crucial role in maintaining the homeostasis of the urinary system, specifically by influencing urethral mucosal thickness, enhancing the tone of the urethral sphincter, and optimizing detrusor muscle function, all of which contribute to the stability of the urinary system (36). As estrogen levels decline, particularly in postmenopausal women, these protective mechanisms may weaken, potentially leading to bladder dysfunction (37). Some studies suggest that both local and systemic estrogen supplementation may help alleviate OAB symptoms (38). Phthalate exposure has also been linked to lipid peroxidation, DNA damage, and cellular dysfunction (39). Emerging research also indicates a close association between gut microbiota and the risk of progression of OAB symptoms (40). Studies have found that phthalate exposure can suppress gut microbiota activity, and these alterations may influence bladder function through the gut-bladder axis (41). Although several potential biological mechanisms have been proposed, we acknowledge that the specific roles of these mechanisms remain to be fully elucidated, necessitating further research to validate these hypotheses.

One of the main strengths of this study is the use of NHANES data, which provides a large, nationally representative sample with detailed information on phthalate exposure and OAB status. Employing a suite of advanced statistical techniques, including multivariable logistic regression, RCS, and BKMR analysis, enabled a thorough and nuanced assessment of these associations. Nonetheless, certain limitations warrant consideration. The cross-sectional nature of this study inherently constrains our ability to infer causality. Additionally, phthalate exposure was gauged using a single urine sample, which may not adequately capture long-term exposure. Furthermore, further classification of OAB into mild, moderate, and severe categories according to the OABSS scoring system may enhance the assessment of the impact of phthalate exposure on OAB of varying severity. Moreover, although many covariates were adjusted for, the possibility of residual confounding factors cannot be completely ruled out. For example, beverage choice seems to be a potential confounder; however, it could not be included in the analysis due to a lack of data. Another limitation of this study is the restricted number of phthalate metabolites analyzed (*n* = 8). Other phthalate metabolites not included in this research may have a more significant impact on OAB. The findings of this study bear substantial public health implications. Considering the pervasive use of phthalates in consumer products, curbing exposure to these chemicals could be a pivotal measure in mitigating OAB risk, especially among vulnerable populations such as women and the older adult. Our findings underscore the imperative for regulatory actions to curtail phthalate exposure, particularly in products predominantly used by populations at heightened risk for OAB. Further investigation is warranted to elucidate the biological mechanisms underpinning the phthalate-OAB association, with a particular focus on the roles of endocrine disruption and inflammation in OAB pathogenesis.

## Conclusions

In conclusion, our study provides some evidence that higher concentrations of specific urinary phthalate metabolites, particularly MBzP and MiBP, are associated with an increased risk of OAB. Given the widespread use of phthalates in consumer products and their ubiquitous presence in the environment, our findings carry significant public health implications. Reducing phthalate exposure, particularly among women and the older adult, may be a crucial step in lowering the risk of OAB and improving overall urinary health. Further research is needed to validate these findings and to explore potential interventions aimed at minimizing phthalate exposure and its impact on bladder function.

## Data Availability

The original contributions presented in the study are included in the article/Supplementary material, further inquiries can be directed to the corresponding author.
