# Supplementary material for: Endocrine disruptors and bladder function: the role of phthalates in overactive bladder
Source: Front Public Health. 2024 Dec 11;12:1493794. doi: 10.3389/fpubh.2024.1493794 (PMC11668814; doi:10.3389/fpubh.2024.1493794)
Supplement: Supplementary file 1 [file Table_1.docx]

Supplementary Material for

**Endocrine Disruptors and Bladder Function: The Role of Phthalates in Overactive Bladder**

**Supplementary table1.** The weighted multivariate logistic regression analysis of the relationship between phthalates exposures and overactive bladder ^a^.

| HMW phthalates | Model 1^b^ (n =6228)  OR (95% CI) | Model 2^c^ (n =6228)  OR (95% CI) | HMW phthalates | Model 3^d^ (n =4451)  OR (95% CI) |
| --- | --- | --- | --- | --- |
| MCNP | 0.984 (0.958 ~ 1.010) | 0.996 (0.977 ~ 1.014) | **MCNP** | 0.997 (0.979 ~ 1.015) |
| Q1 (<1.1) | Reference | Reference | Q1 **(**<1.2**)** | Reference |
| Q2 (1.1–2.7) | 1.144 (0.818 ~ 1.599) | 1.129 (0.801 ~ 1.591) | Q2 **(**1.2–2.8**)** | 1.096 (0.771 ~ 1.558) |
| Q3 (>2.7) | 0.952 (0.671 ~ 1.350) | 1.184 (0.826 ~ 1.695) | Q3 **(**>2.8**)** | 1.087 (0.752 ~ 1.573) |
| MCOP | 0.999 (0.997 ~ 1.001) | 1.000 (0.999 ~ 1.002) | **MCOP** | 1.001 (0.999 ~ 1.003) |
| Q1 (<5.2) | Reference | Reference | Q1 **(**<5.5**)** | Reference |
| Q2 (5.2–17.47) | 1.298 (0.923 ~ 1.825) | 1.341 (0.945 ~ 1.902) | Q2 **(**5.5–18.6**)** | 1.260 (0.881 ~ 1.801) |
| Q3 (>17.47) | 1.139 (0.802 ~ 1.619) | 1.387 (0.967 ~ 1.989) | Q3 **(**>18.6**)** | 1.318 (0.911 ~ 1.905) |
| MECP | 1.000 (0.996 ~ 1.003) | 1.001 (0.997 ~ 1.005) | **MECP** | 1.000 (0.995 ~ 1.005) |
| Q1 (<6.5) | Reference | Reference | Q1 **(**<6.4**)** | Reference |
| Q2 (6.5–14.7) | 1.267 (0.891 ~ 1.801) | 1.313 (0.915 ~ 1.884) | Q2 **(**6.4–14.6**)** | 1.294 (0.895 ~ 1.869) |
| Q3 (>14.7) | 1.353 (0.957 ~ 1.915) | **1.483 (1.038 ~ 2.117) ^∗^** | Q3 **(**>14.6**)** | 1.347 (0.935 ~ 1.942) |
| MCPP | 1.000 (0.999 ~ 1.002) | 1.001 (0.999 ~ 1.003) | **MCPP** | 1.001 (0.999 ~ 1.003) |
| Q1 (<0.9) | Reference | Reference | Q1 **(**<0.9**)** | Reference |
| Q2 (0.9–2.4) | 1.205 (0.858 ~ 1.692) | 1.211 (0.855 ~ 1.717) | Q2 **(**0.9–2.4**)** | 1.195 (0.837 ~ 1.708) |
| Q3 (>2.4) | 1.053 (0.744 ~ 1.490) | 1.231 (0.862 ~ 1.760) | Q3 **(**>2.4**)** | 1.177 (0.817 ~ 1.696) |
| MBzP | 1.003 (0.998 ~ 1.009) | **1.008 (1.003 ~ 1.013) ^∗∗^** | **MBzP** | **1.006 (1.001 ~ 1.012) ^∗^** |
| Q1 (<2.2) | Reference | Reference | Q1 **(**<2.1**)** | Reference |
| Q2 (2.2–6.4) | 1.257 (0.885 ~ 1.786) | 1.361 (0.949 ~ 1.950) | Q2 **(**2.1–6.3**)** | 1.247 (0.863 ~ 1.801) |
| Q3 (>6.4) | 1.323 (0.933 ~ 1.877) | **1.761 (1.227 ~ 2.527) ^∗∗^** | Q3 **(**>6.3**)** | **1.509 (1.041 ~ 2.186) ^∗^** |
| LMW phthalates |  |  | **LMW phthalates** |  |
| MEP | 1.000 (1.000 ~ 1.000) | 1.000 (1.000 ~ 1.000) | **MEP** | 1.000 (1.000 ~ 1.000) |
| Q1 (<19.7) | Reference | Reference | Q1 **(**<19.5**)** | Reference |
| Q2 (19.7–68.6) | 1.119 (0.786 ~ 1.594) | 1.137 (0.792 ~ 1.634) | Q2 **(**19.5–67.9**)** | 1.147 (0.792 ~ 1.662) |
| Q3 (>68.6) | 1.363 (0.971 ~ 1.915) | 1.363 (0.962 ~ 1.930) | Q3 **(**>67.9**)** | 1.341 (0.939 ~ 1.916) |
| MBP | 1.000 (0.998 ~ 1.003) | 1.000 (0.998 ~ 1.003) | **MBP** | 1.000 (0.998 ~ 1.003) |
| Q1 (<6.3) | Reference | Reference | Q1 **(**<6.1**)** | Reference |
| Q2 (6.3–15.4) | 1.332 (0.936 ~ 1.894) | 1.346 (0.938 ~ 1.931) | Q2 **(**6.1–15.3**)** | 1.337 (0.925 ~ 1.933) |
| Q3 (>15.4) | 1.395 (0.984 ~ 1.976) | **1.457 (1.019 ~ 2.083) ^∗^** | Q3 **(**>15.3**)** | 1.430 (0.990 ~ 2.064) |
| MiBP | 1.001 (0.997 ~ 1.006) | **1.005 (1.001 ~ 1.009) ^∗^** | **MiBP** | **1.004 (1.001 ~ 1.008) ^∗^** |
| Q1 (<4.9) | Reference | Reference | Q1 **(**<4.8**)** | Reference |
| Q2 (4.9–11.9) | 0.984 (0.693 ~ 1.399) | 1.118 (0.779 ~ 1.603) | Q2 **(**4.8–11.9**)** | 1.054 (0.728 ~ 1.526) |
| Q3 (>11.9) | 1.223 (0.875 ~ 1.708) | **1.588 (1.123 ~ 2.245) ^∗∗^** | Q3 **(**>11.9**)** | **1.555 (1.088 ~ 2.224) ^∗^** |

Abbreviations: ^a^: individuals with a total score of ≥5 were classified as having OAB. ^b^ Model 1: no adjusted. ^c^ Model 2: adjusted for age, sex. ^d^ Model 3: Adjusted for all covariates. ^∗^*p*<0.05, ^∗∗^*p*<0.01, ^∗∗∗^*p*<0.001. Bold values indicate statistical significance.
